# The Life of Edward Jenner, M.D. &c.

**Published:** 1827-07

**Authors:** 


### VIII.

*The Life of Edward Jenner, M.D. L.L.D. F.R.S. Physician Extraordinary to the King, &c. &c. with Illustrations of his Doctrines, and Selections from his Correspondence.* By JOHN BARON, M.D. F.R.S. Octavo, pp. 608. London, Colburn, 1827.

THE grave has now closed over the author of vaccination. Small-pox was certainly one of the severest scourges that ever infested humanity; it was worse than the plague itself, since that calamity either destroyed its victim or permitted him to escape; while this, if it failed to extinguish life, too often either deprived the unhappy sufferer of vision, or rendered his face unseemly to the sight of strangers, and heart-rending to friends. Inoculation was undoubtedly of great utility, it was a counterpoise, and not an insignificant one, to affliction, deformity, and death; yet still it occasionally failed, and took away life from the innocent and healthy being that had been consigned to its protection. To inoculation, vaccination followed, which is justly characterized to be one of the greatest improvements ever made in the practice of medicine. If vaccination had only succeeded once in twenty cases, the benefits of it would have been considerable, but they become nearly incalculable, when, beyond all dispute, it is successful in guarding the constitution from variolous infection in the plurality of instances. Again, had vaccination been a disgusting or dangerous experiment, the advantages of it might have been depreciated: but, when it is indubitably the reverse, every one must acknowledge the almost universal blessing which it has bestowed upon the human race. "Envy will merit as its shade pursue;" censure is the tax that talent pays to the world, since the author of every discovery or improvement of importance has been vilified in his turn, and his right to them either denied or controverted. Such is the perversity of mankind, such the injustice practised towards genius, that the laurel crown is first withheld from him that has fairly won it, and when meekly worn, it is attempted to be plucked from his brows by base and jealous hands. Yet is it highly honourable to the medical profession, that, although the small-pox and its direful consequences were a source of no inconsiderable emolument, they received and propagated vaccination with singular ardour and disinterestedness, and incontestibly demonstrated, that, in them, the love of science was superior to the desire of gain. Unquestionably, the vaccine inoculation had its opponents, but they were insignificant in rank and numbers, and could boast of no respectability, except what might attach to the names of Ingenhouse, Moseley, Rowley and Birch. During the last sixteen years, the practice of vaccination has been fixed on a permanent basis; its feeble enemies, feeble even in mischief, have sunk into oblivion; and though it has not, like the test of a literary reputation, survived its century, little doubt can now be entertained of its eternal success. Vaccination has subtracted one from the sum of human miseries; it has not only guarded mankind against a most pestilential disease, but preserved beauty, the charm which either graces intellect, or atones for its deficiency, from a deformity that was sometimes worse than death.

Who Edward Jenner was, where he flourished, what was his character, what his pursuits and acquirements, what the mark of his genius, how his juvenile mind, in a happy moment, unalterably fixed itself on the popular tradition of his native vale, and how, by acute investigation, and accurate experiments, he attained, amid anxiety and disappointment, calumny and opposition, the darling object of his soul, and exalted himself among the benefactors of his species, are questions which will be often agitated. Dr. Baron, of Gloucester, appears, with singular propriety, as the biographer of Dr. Jenner. He knew him most intimately, and had known him long; he admired his virtues, and was zealous for his fame; and, independently of all private feelings, he was solicited to write, and stimulated to exertion by having the necessary papers of the deceased consigned to his inspection. He lived not at a distance, or in a distant age; he had not common traditional report to depend upon; or vague information to guide him, which might have been, and frequently is, clouded by prejudice, obscured by ignorance, and misrepresented by malevolence. To conclude, he had, and has had, abundant fair play:—Personal knowledge, long intercourse, unreserved friendship, local residence, satisfactory documents, and numerous relatives, and warm friends, anxious to support him in his undertaking, and ready to cheer him in his progress. If this be not a royal road to biography, words have lost their meaning; and, unlike royal roads, it has been trodden by a writer that deserved it.

The first one hundred and twenty pages of this handsome volume, which is ornamented by a likeness of the author of vaccination, printed on fine paper, and dedicated to the King, are devoted to the private life of Dr. Jenner, up to 1798, when he published his "Inquiry," including letters from John Hunter to him from 1773 to 1783; and the remaining four hundred and eighty-eight are principally assigned to the early history of vaccination, and to disquisitions on it, small-pox, and the peculiar opinions modestly and elegantly advanced on these subjects by the deceased, and which are now revived, and maintained to be correct by his biographer. The publication of the first part, (the present work) without waiting for the completion of the second, says Dr. Baron, in his introduction, seemed to be expedient, both to the executors of Dr. Jenner and to myself, and other reasons concurred to give strength to this decision. When this second part appears, we shall not fail to notice it; and, meanwhile, overlooking, as we necessarily must do, all vaccine and variolous topics, we will attempt to compose, from the materials now before us, a brief and faithful biographical article. Such an article cannot be otherwise than interesting, since where is the reader who does not wish to know more or less of the life, character, and abilities of the man that rendered such a signal service, not only to his country, but the world; not only to the millions now living, but to millions yet unborn?

Edward Jenner was born in the vicarage at Berkeley, in Gloucestershire, on the 17th of May, 1749, and was the third son of the Reverend Stephen Jenner, A.M. of Oxford, rector of Rockhampton, and vicar of Berkeley, by the daughter of the Rev. Henry Head, of an ancient and respectable family in Berkshire. The father of Jenner also possessed considerable landed property, the family being of great antiquity in Gloucestershire, and the neighbouring county of Worcester. He was unfortunately consigned to the tomb, soon after the birth of Edward, in the year 1754, at the age of fifty-two. This heavy loss to Edward was partly alleviated by his eldest brother, Stephen, a clergyman; and he had likewise another brother, named Henry, of the same profession.

From him sprang the Rev. George C. Jenner, and Mr. Henry Jenner, who afterwards assisted their uncle Edward in his pursuits. Jenner had three sisters, Mary, Sarah, and Ann, who was married to the Rev. Wm. Davies, and gave birth to three sons.

Jenner, when about the age of eight years, was put to school at Wotton-under-Edge, under the Rev. Mr. Clissold. He was next placed at Cirencester, under the Rev. Dr. Washbourn, where he made a respectable proficiency in the classics, contracted friendships which continued through life, and displayed his taste for natural history. Having finished his education, he was apprenticed to Mr. Ludlow, an eminent surgeon and apothecary in Sodbury, a very small town, twelve or fourteen miles distant from Bath and Bristol. When his term was expired, "he went to London, to prosecute his professional studies under the direction and instruction of the celebrated John Hunter, in whose family he resided for two years, a favourite pupil." At this period, Jenner was twenty-one, and Hunter 42 years of age, and it is easy to conceive how the master-mind of the latter would incite the intellectual and physical powers of the former. After completing his studies, he retired from the house of Mr. Hunter, and continued with him an uninterrupted epistolary correspondence. Jenner set a high value on that gentleman's letters, and preserved them with great care. We have read them with much pleasure. It is refreshing to contemplate the physiologist as a letter-writer; they are eminently characteristic of his mind; relate chiefly to subjects of natural history; and evince that ardour for knowledge which death could only extinguish. The friend and correspondent of Hunter, a proof he was no common man, immediately after he left London, commenced practice, as a surgeon and apothecary, in his native village of Berkeley, sixteen miles from the city of Gloucester, and took up his residence with his brother Stephen. His talents and conduct soon gained for him numerous friends and a rapidly increasing practice. He frequently took long rides on horseback, either contemplating the beauties of nature, or revolving in his mind the wonders of art and science. In these rides, he was often accompanied, for twenty or thirty miles, by friends that esteemed his character and loved his society. His recreations consisted, at this time, of visiting agreeable families, with whom he was always a favourite; and in the cultivation of polite literature. Occasionally he sought an acquaintance with the muses; his imagination, indeed, was vivid, and he enjoyed a peculiar facility, even in conversation, of clothing his observations in the gay and lively colours of poetry. In this he was assisted by his knowledge of the economy of plants and animals, and his vigilant attention to all the varied forms and properties of surrounding objects. Dr. Baron has presented to his readers "a few of Jenner's poetical *jeux d'esprit.*" In our opinion, they display talent, in conjunction with a light, elegant, and playful fancy. A delineation so characteristic as the following ought not to be omitted; it was given to the biographer by the late Mr. Edward Gardner, a clever and well educated man, who had been the schoolfellow of the unfortunate Chatterton, and the cordial friend of the deceased for more than forty years. "His height was rather under the middle size, his person was robust, but active, and well formed. In his dress he was peculiarly neat, and every thing about him showed the man intent and serious, and well prepared to meet the duties of his calling. When I first saw him, it was on Frampton Green. I was somewhat his junior in years, and had heard so much of Mr. Jenner, of Berkeley, that I had no small curiosity to see him. He was dressed in a blue coat and yellow buttons, buckskins, well-polished jockey boots, with handsome silver spurs, and he carried a smart whip, with a silver handle. His hair, after the fashion of the times, was done up in a club, and he wore a broad-brimmed hat. We were introduced on that occasion, and I was delighted and astonished. I was prepared to find an accomplished man, and all the country spoke of him as a skilful surgeon and a great naturalist; but I did not expect to find him so much at home on other matters."

Mr. Jenner on all occasions, promoted good company, and good discourse, as the sinews of virtue. He was especially fond of music, and was a member of a catch club, which met at Cam. He could also play on the violin and flute, and formed select musical parties, wherein he was occasionally a performer. Like most wise men, he had a particular dislike to cards. Thus did Jenner pass his time, in active professional engagements, scientific pursuits, and engaging society; and he was instrumental in forming two medical and convivial societies, to which he was a lively and able contributor. In one of them, he often recurred to the prophylactic powers of the cow-pox, until, at length, the subject became so distasteful to his companions, that they threatened, poor short-sighted and ill-judging mortals, to expel him from their meetings. About 1775 or 1776, Mr. Jenner was disappointed in love, and, for several years, suffered most severely. Two of his letters to his friend Gardner, written in 1783, seem to refer to this interesting subject. In one he says:—"I am jaded almost to death, my dear Gardner, by constant fatigue: that of the body I must endure; but how long I shall be able to bear that of the mind, I know not. Still the same dead weight hangs upon my heart. Would to God it would drag it from its unhappy mansion! then with what pleasure could I see an end of this silly dream of life." Again, on the 8th of April of this year, he writes thus to the same friend:—"As for myself, the same stream of unhappiness is still flowing in upon me; its source seems inexhaustible; but there is a soothing consolation in it; all little disquietudes are sunk or washed away. I feel their influence no more." But, though broken in spirit by a cause at which no man need blush, since it springs from the finest feelings of the human heart, he attended to his profession, pursued his studies, and resolved that, if he did die, it should be "with harness on his back." Fortunately for a grateful world he did not die. Afterwards he performed several experiments; happily explained the apparently unnatural conduct of the cuckoo, that almost invariably commits her offspring to the care of a foster parent; and, finally, he proved, what no wise or observing person ever doubted, that a man may love twice, for, on the sixth of March, 1788, he was united in marriage to Miss Catharine Kingscote, a lady on whom his affections had long been fixed. Elegant in her manners, accomplished in her mind, vigorous in her understanding, and descended from an ancient and respectable family, she made him completely happy. But she had for a considerable time been an invalid, and never enjoyed robust health. On the 24th of January, 1789, this lady presented to her husband his eldest son Edward, to whom John Hunter was godfather. This gentleman's last letter to Jenner was dated on the 12th of August, 1793, about two months before he suddenly expired in St. George's Hospital. It is written with his usual powers. His sincere friend unceasingly lamented his death, and always termed him "the dear man."

The fatigues of an extensive general practice having become irksome, Mr. Jenner, now in his forty-third year, resolved to confine himself to medicine, and obtained in 1792 a degree of Doctor of Physic from the ancient university of St. Andrew's. Toward the conclusion of 1794, he was attacked with typhus fever, which nearly proved fatal, and its effects continued for a considerable time. In 1797 Dr. Jenner had almost arranged every thing for the publication of his Inquiry. His attention was called forcibly, and for the first time, to the nature of cow-pox, while he was a youth. When an apprentice in Sodbury, a young woman applied for advice; the subject of small-pox was mentioned in her presence; she immediately observed, "I cannot take that disease, for I have had cow-pox." This incident fixed itself on the mind of Jenner, and laid the foundation of his future fame. Such are the trifles, which, sometimes remembered and sometimes forgotten, determine the bent of individual genius, and contribute to the most important results. In 1770 Jenner communicated this circumstance to John Hunter, who, never damping the ardour of a pupil, replied, "Don't *think*, but *try*; be patient, be accurate." He made known Jenner's opinions, and the traditions in Gloucestershire, both in his lectures, and to his friends in conversation; and other lecturers, on his authority, mentioned them to their pupils. In 1780 Jenner was enabled, after much study and enquiry, to explain many of the perplexing obscurities and contradictions, with which the subject was embarrassed. On the 14th of May, 1796, he, for the first time, vaccinated, and with success, the arms of James Phipps, a healthy boy of about eight years of age, with fluid taken from the hand of Sarah Nelmes, who had been infected by her master's cows. Dr. Jenner published his Inquiry in June, 1798. It contained seventy-three or four quarto pages, and was dedicated to the late Dr. Parry of Bath, who had already been indebted to him for the ground-work of his book on angina pectoris. He visited London in April, 1798, and was received with kindness and respect. From this period, the vaccine inoculation, in spite of real enemies and pretended friends, besotted admirers and ignorant opponents, gradually expanded itself over various parts of the globe; honours flowed in upon the author of it; and he enjoyed the happiness of a man, that knew he had laboured well, and successfully. Strange to relate, yet every age can furnish a parallel, his native county of Gloucester, instead of being the first to enter the lists of generosity to reward her distinguished son, could only raise for him a small service of plate, while a British House of Commons, not indeed then led by the master mind of a Canning, coldly voted, by a majority of three, the sum of ten thousand pounds for his services to humanity. In a pecuniary point of view, it is obvious that Jenner could scarcely have been a gainer by vaccination, since he had incurred great expenses in postage, travelling, and long residences in London, combined with anxiety of mind, intense occupation, a neglect of his private affairs, and an unavoidable injury to a lucrative medical practice. These circumstances, said he to a committee of the House of Commons, have exposed me to a serious evil; and I never could have persevered, to the obvious injury of my family, had I not been buoyed up by a confidence in the generosity of my country.

Having adverted to the peculiar opinions of Dr. Jenner, it may be proper to record them. He always maintained the grease of horses to be "the source of small-pox;"* that "small-pox and cow-pox were modifications of the same distemper,"† hence his name of Variolæ Vaccinæ; and he actually produced the latter in the human subject by the direct introduction of equine virus,‡ and with subsequent immunity from variolous infection. The first and second propositions are, it must be acknowledged, startling; they are apparently irreconcilable with the common sense of mankind, yet they were long considered, and finally declared by a physician, who had much at stake, and who was certainly of no inferior mind, and of no insignificant judgment. We ought not, therefore, with a dogmatism, which seldom springs from a philosophical understanding, to assert them to be false, without due inquiry and laborious investigation, because they may surprise us by their novelty, shock our prejudices, or clash with our pre-conceived opinions. Dr. Baron attempts, with no little zeal and some learning, to demonstrate these propositions to be correct. For this purpose, he has striven to trace small-pox to nearly 1400 years before the Christian era; has eagerly enlisted the Old Testament into his service, and resolutely impressed Philo, the learned Jew: he has also, with singular enthusiasm, flanked himself with the testimonies of Orosius, Dyonysius of Halicarnassus, Homer, and Livy, and Virgil; not content with such able forces, he has fronted his cause with Herodian, Thucydides, Herodotus, Hippocrates; and, that nothing might be wanting, which anxiety and care could supply, he has prudently brought up his rear by Julius Capitolinus, Cyprian, Eusebius, Zosimus, Procopius, Evagrius, and Paulus Diaconus. But a painful acknowledgment, on our part, remains to be made; we are still unconvinced; and cannot permit acquiescence to precede conviction,

We have now continued the life of Dr. Jenner to his fifty-first year, for that of a physician, or man of letters, is soon told; and nothing can be left but an account of his declining years, his illness, death, and

* Baron's Life of Jenner, p. 135. † Ibid. p. 162. ‡ Ibid. p. 148.

surviving family; and here we are obliged to stop, for here our biographical guide forsakes us to state the progress of vaccination from the formation of the Royal Jennerian Society to the departure of the expedition from Spain, with which his volume concludes. It may be said of human talents, and of human actions, as Solon affirmed to Crœsus of human happiness, that we should wait until the life be terminated, before we pronounce decisively upon them. With Edward Jenner that awful period has arrived; and we, who are neither his friends nor biographers, have that duty to perform; and we prefer to perform it now and prior to the appearance of the second volume, for unanswerable reasons. Panegyric, general and indiscriminate, is a violation of biographic truth, and is oftener the sign of a weak than of a strong mind. While attempting to ascertain the character, the intellect, and the acquirements and performances of an individual, however amiable and regretted, the interests of truth, of justice, and of science, should be rigorously observed. It is not a question of mere kindness, or of cold calculation, but one of paramount importance, which comprises the sacred rights of the dead, and involves the momentous ones of the living. In estimating with scrupulous accuracy the exact degree of merit that belongs to a man, not now of this world, his birth, his education, his advantages, and his every circumstance, local and fortuitous, are to be rigidly weighed, and carefully placed in the opposite scale to that which contains what he has acquired, or what he has accomplished. The ascension of intellect, in whatsoever way it springs forth, can only be fairly measured from the altitude whence that intellect winged its flight. He who reaches the sky from a lofty mountain sinks before him that has attained the same sky by an ascent out of the lowly vale. These are, it is presumed, indisputable positions. Equally indisputable is it that Edward Jenner's ascension was from the lofty mountain. He was born, educated, and lived under academic bowers: he was the son of a beneficed clergyman of considerable landed property, and of ancient family; he was respectably connected by his mother; and he was enabled by his pecuniary resources, to live for two years, no common lot and no mean advantage, as a favourite pupil in the house of John Hunter. Under the most flattering auspices he commenced surgical and medical practice in his native village, of which his father had been vicar, where his brother was a much esteemed clergyman, and where, in consequence of his family and fortune, he was instantly received into genteel society. How different then was Jenner's fate from that of the man, whom a proud aristocracy disdains to own; the son of humble parents; self-taught; hastily educated in the metropolis; and who, at his entrance into life, as a general practitioner, begins upon nothing; unknown, unfriended, unrecommended; shunned by men who immure themselves within the bastile of their rank; who has to provide for the day that is passing over him; and who well knows, since an unfeeling world stamps it hourly on his recollection, that with him it will not be dignified ease, or literary leisure, but prosperity, or adversity, expatriation, or a prison. If such a man, and the picture thus feebly drawn can find a thousand originals, should shake off all these appalling evils, and rise, as many have risen, to distinguished eminence, how greatly would he transcend the individuals who, Jenner like, were born under a happy star, on whom fortune always smiled, and who never knew, because they never felt, how "slow rises worth, by poverty depressed." If the preceding observations be just, it will follow that the acquirements, performances, and success of Dr. Jenner present nothing remarkable, with the exception of vaccination. Even in this his polar star shone brightly, for had he belonged to, and practised in, a distant county, it is undeniable that the subject of cow-pox would never have engaged his attention. But here, indeed, his merit is pre-eminent, and above all praise;—he took up a popular tradition; he subjected it to experiment; he overcame difficulties; reconciled contradictions; elucidated obscurities; converted uncertainty into certainty; and exalted a dubious truth to the stability of science, while every other medical man, enjoying equal opportunities, had either neglected, or depreciated enquiry; and, as far as in his power laid, had either sought to extinguish, or striven to cloud a glimmering light, that would have conducted its preserver to immortality.

Dr. Jenner has been termed by his biographer, and other authors, the discoverer of the protecting powers of cow-pox. This cannot be acceded to; it is at variance with truth. Jenner is so rich in real merit that his admirers ought to disclaim for him an honour to which he is not entitled. How can he be called a discover, when, independently of the well known tradition of his native county, his youthful mind was irrevocably directed to that subject on hearing a young woman emphatically observe, in his master's house at Sodbury, "I cannot take small-pox for I have had cow-pox?" "This incident," says Dr. Baron,* "rivetted the attention of Jenner. It was the first time that the popular notion, which was not at all uncommon in the district, had been brought home to him with force and influence. Most happily the impression which was then made was never effaced." Surely this statement must for ever prevent Dr. Jenner from ranking as a discoverer. Harvey discovered the circulation of the blood; but if that had been already known in his own county, though expressed in vernacular language, and if a young man had pointedly fixed the boyish mind of the future physiologist on such a momentous circumstance, and if, in the prime of manhood and by scientific experiments, he had demonstrated it to be correct, and promulgated the particulars to the world, would he then have been pronounced a discoverer? No! He would have been designated the demonstrator, and promulgator of a long and partially known, but much neglected, popular truth. Precisely similar are the irrefragable claims of Jenner; they are sufficient to gratify human ambition; they need not that to which he has no legitimate pretension. Posterity, always just, will never recognize Edward Jenner as a discoverer, yet will it assign to him an exaltation scarcely less enviable; and acknow-

* Life of Jenner, p. 122.

ledge him to be one of the greatest benefactors of the human race, a benefactor, not limited by time and space, but interminable and everlasting. From contemporary writers other ages must learn his character, and if they be swayed by the solemn dictates of truth, they will portray him amiable in disposition, spotless in reputation, and accomplished in mind;—playful yet serious, gay yet studious, condescending yet dignified.

We lay down the pen, and congratulate the friends and executors of the deceased on having selected Dr. Baron for his biographer, who has performed his delicate and arduous office, as far as he has proceeded, with care, fidelity, and judgment, and written with candour, spirit, and talent.*

